# Real-World Data on the Use of Nivolumab Monotherapy in the Treatment of Advanced Renal Cell Carcinoma After Prior Therapy: Final Results from the Non-Interventional NORA Study

**DOI:** 10.3390/cancers18132075

**Published:** 2026-06-26

**Authors:** Marc-Oliver Grimm, Viktor Grünwald, Harald Müller-Huesmann, Philipp Ivanyi, Martin Schostak, Eyck von der Heyde, Wolfgang Schultze-Seemann, Holger Schulz, Martin Bögemann, Stefan Wolfgang Grötzinger, Luis Vaz, Martin Herber, Jens Bedke

**Affiliations:** 1 Department of Urology, Jena University Hospital, Friedrich-Schiller University Jena, 07747 Jena, Germany; 2 Comprehensive Cancer Center Germany (CCCG), 07747 Jena, Germany; 3 Clinic for Medical Oncology and Clinic for Urology, West-German Cancer Center Essen, Essen University Hospital, 45147 Essen, Germany; 4 Department of Internal Medicine, Hematology and Oncology, St Josef Brothers Hospital, 33098 Paderborn, Germany; 5 Clinic Department of for Hematology, Hemostaseology, Oncology and Stem Cell Transplantation, Interdisciplinary Oncologic Outpatient Clinic (OIA), Hannover Medical School, 30625 Hannover, Germany; 6 LOGICURO GmbH, 14482 Potsdam-Babelsberg, Germany; 7 Department of Urology, Urooncology, Robot-Assisted and Focal Therapy, Magdeburg University Hospital, 39120 Magdeburg, Germany; 8 Practice for Oncology Raschplatz, 30161 Hannover, Germany; 9 Department of Urology, Freiburg University Hospital, 79106 Freiburg, Germany; 10 Practice for Medical Oncology and Hematology, 50226 Frechen, Germany; 11 Department of Urology, Münster University Hospital, 48149 Münster, Germany; 12 Bristol Myers Squibb Germany, 80636 Munich, Germany; 13 Bristol Myers Squibb UK, Uxbridge UB11 1AF, UK; 14 Department of Urology and Transplant Surgery, Stuttgart Hospital, 70174 Stuttgart, Germany

**Keywords:** immunotherapy, real-world data, effectiveness, nivolumab, safety

## Abstract

**Background/Objectives**: Nivolumab monotherapy is a standard of care in previously treated advanced renal cell carcinoma (aRCC) and was approved based on the results of the randomized clinical trial Checkmate-025. The non-interventional study (NIS) NORA collected data on the effectiveness and safety of nivolumab monotherapy in real-world clinical routine. Its final, long-term follow-up data are presented here. **Methods**: NORA was a prospective, multicentre NIS recruiting at 54 German sites, evaluating the effectiveness and safety of nivolumab monotherapy in pre-treated patients with aRCC. Endpoints included overall survival (OS), progression-free survival (PFS), objective response rate (ORR), duration of response (DOR), safety, and patient-reported outcomes (PROs). **Results**: A total of 232 patients were eligible. Of the patients, 15% had favourable, 58% had intermediate, and 15% had poor risk according to the International Metastatic RCC Database Consortium. Of the patients, 77% received nivolumab as second-line, 15% as third-line, and 8% as ≥fourth-line therapy. With a median long-term follow-up of 76 months (minimum 62 months), median OS was 22.2 months (95% confidence interval [CI] 16.5–25.9) and median PFS was 4.1 months (95% CI 3.2–5.4). The ORR was 21% with a median DOR of 27.9 months (95% CI 15.9—not evaluable). Of the patients, 47% and 16% had treatment-related adverse events of all grades and of grades 3–4, respectively. One patient died from autoimmune hepatitis related to treatment. PROs did not reveal any new signals. **Conclusions**: The long-term follow-up of NORA confirms that nivolumab monotherapy is an effective and safe therapy in patients with aRCC after prior therapy. With comparable follow-up times, our real-world data were not substantially different from the final long-term results of the pivotal Checkmate-025 study.

## Simple Summary

Nivolumab is a widely used treatment for patients with advanced kidney cancer who have already received prior therapies. We report final data from the NORA study on the effectiveness and safety of this therapy in “real-world” clinical practice. These data are very mature with long treatment and observation periods. A total of 232 patients participated in the study. On average, patients lived for 22 months after starting nivolumab, and they lived without their cancer progressing for 4 months. Of all patients, 21% responded to nivolumab. Nearly half of the patients experienced side effects related to the treatment, and 16% had severe treatment-related side effects. Our results were similar to those of the CheckMate-025 trial that led to approval of this therapy in 2016. Thus, the NORA study confirmed that nivolumab monotherapy is an effective and safe treatment option for patients with advanced kidney cancer who have progressed after prior anti-angiogenic therapy.

## 1. Introduction

Nivolumab, a fully human antibody, is an immune checkpoint inhibitor targeting programmed cell death protein 1 (PD-1). It prevents the interaction with the PD-1 ligand PD-L1 and restores the otherwise downregulated antitumor immune response [1,2]. Since its regulatory approval by the European Medicines Agency (EMA) in 2016, nivolumab as monotherapy has become an integral part of the treatment landscape of advanced renal cell carcinoma (aRCC) after prior therapy [3,4]. The results of the randomized, open-label, phase 3 clinical CheckMate-025 clinical trial were pivotal. When applied in patients with aRCC and one or two prior anti-angiogenic therapies, nivolumab (3 mg/kg, once every two weeks, intravenously) was shown to improve overall survival (OS), objective response rates (ORR) and safety as compared to a former second-line standard of care, everolimus (10 mg once a day, orally) [5]. These advantages were maintained at a prolonged median follow-up of 72 months (minimum 64 months), with regard to median OS (25.8 months [95% confidence interval (CI), 22.2–29.8 months] vs. 19.7 months [95% CI, 17.6–22.1 months]; hazard ratio [HR] 0.73 [95% CI, 0.62–0.85]; *p* < 0.0001), ORR (23% vs. 4%; *p* < 0.001), and reduced grade 3 or 4 adverse events (21% vs. 37%) [6].

However, the strict selection criteria in randomized clinical trials (RCTs) do not fully reflect the diversity of patients in clinical routine including different histologies [5,7]. Studies involving real-world populations may fill this gap and provide important insights into the effectiveness and safety of therapies in everyday clinical practice [8]. The non-interventional study NORA (**N**iv**O**lumab in **R**enal cell c**A**rcinoma) is a prospective, observational, multicentre study evaluating nivolumab monotherapy after prior therapy (second- or later lines), and nivolumab in combination with ipilimumab as a first-line treatment in patients with aRCC. We have already published the primary study endpoint of the nivolumab monotherapy cohort with minimum and median follow-up times of 24 months and 37 months, respectively [9]. With earlier diagnosis of cancer, more effective treatment options, and the resulting longer survival of cancer patients, the assessment of long term benefits, late recurrence and late adverse effects is becoming increasingly important [10]. In addition, subsequent therapies and therapy-free intervals are attracting growing attention [6,11,12,13]. This article presents final data from the completed nivolumab monotherapy cohort with 3 years of additional minimum follow-up as compared to the formerly reported data, which are comparable to the long-term follow-up period of CheckMate-025. It focuses on clinical outcomes, patient characteristics and the safety of patients with aRCC receiving nivolumab monotherapy after prior therapy in routine clinical practice in Germany—either overall or by relevant subgroups.

## 2. Patients and Methods

### 2.1. Study Design and Participants

NORA was a prospective, observational, multicentre non-interventional study (NIS) that recruited patients at 54 sites in Germany. It collected real world data of patients with advanced/metastatic RCC who—in the cohort we report on herein—started nivolumab monotherapy after prior therapy in line with the German market authorization. It was mandatory that the treating physician’s decision to start treatment with nivolumab monotherapy for advanced/metastatic RCC was taken independently and before the decision to invite the patient to participate in the study. The study design as well as inclusion and exclusion criteria have been reported previously [9]. In brief, eligible patients were ≥18 years old and diagnosed with histo- and cytologically confirmed advanced/metastatic RCC with clear cell (ccRCC) or non-clear cell (nccRCC) histology based on local pathology reports. The decision to start nivolumab monotherapy after prior therapy (i.e., at least second-line) had been made, but the first dose of treatment had not yet been administered.

The trial was approved by all relevant ethics committees and regulatory authorities and conducted in compliance with the International Society for Pharmacoepidemiology Guidelines for Good Pharmacoepidemiology Practices and in respect of the Declaration of Helsinki [14]. Before entering the study, all patients signed informed consent forms. NORA is registered with ClinicalTrials.gov, NCT02940639. It was supported by the AUO study group (Arbeitsgemeinschaft Urologische Onkologie, AN 47/17).

### 2.2. Objectives

The primary endpoint was OS in the overall population and in relevant subgroups (e.g., non-clear cell histology, risk groups according to the International Metastatic RCC Database Consortium (IMDC) [15], Karnofsky performance score (KPS) < 70, age ≥ 65 years). Secondary objectives included estimation of progression-free survival (PFS), best overall response (BOR), objective response rate (ORR), disease control rate (DCR), and duration of response (DOR) in the overall population and relevant subgroups (see above). Progression was assessed by the investigator, based on clinical or, if available, on imaging evaluation according to the Response Evaluation Criteria in Solid Tumors (RECIST version 1.1) [16].

The treatment-free interval was defined as the time between discontinuation of the study medication and (i) initiation of subsequent systemic anticancer treatment or (ii) the date on which a patient was last known to be alive among patients who never received subsequent systemic anticancer treatment.

Incidences, severity, and management of adverse events (AE) and treatment-related AE (trAE) were evaluated and graded according to the National Cancer Institute Common Terminology Criteria for Adverse Event (NCI CTCAE) grading system, version 4.0 [17]. Patient-reported outcomes (PRO) were assessed at baseline, week six and months three, six, nine, 12, 18, 24, 36, 48 and 60 with the questionnaires Functional Assessment of Cancer Therapy—Kidney Symptom Index-19 (FKSI-19) [18] and European Quality of Life 5 Dimensions 3 Level Version (EQ-5D-3L) [19].

### 2.3. Statistical Analysis

All patients who fulfilled the study inclusion criteria were considered in the data set for analysis. Since NORA was descriptive, no formal hypotheses were tested. The effectiveness of nivolumab was not compared to other treatments. Instead, patient and clinical characteristics were summarized using descriptive statistics to provide context for the observations of patients treated with nivolumab in real life. Statistical analysis was performed using SAS (version 9.4, SAS Institute, Cary, NC, USA).

OS and PFS were estimated using the Kaplan–Meier method. Medians and two-sided 95% CIs were calculated. The index date (day 0) corresponds to the day of the first dose of nivolumab treatment. Patients were censored at their last record or assessment for those lost to follow-up, or at the date of enrolment in a clinical trial. ORR, DCR, BOR are presented as rates. Subgroup analyses are performed as complete cases.

TrAEs were coded by severity grade according to NCI-CTCAE version 4.0 and reported as preferred terms using descriptive statistics. PROs were assessed as mean change from baseline.

## 3. Results

### 3.1. Baseline Characteristics

In total, 232 patients were eligible and enrolled from October 2016 to December 2018. Herein, we report the final study analysis with a minimum follow-up of 62.2 months (median follow-up 75.6 months).

Patients’ baseline characteristics are summarized in Table 1. Of the patients, 166 (72%) were male and 66 (28%) were female. Median age was 71 years (range 44–86) and a KPS ≥ 70 was documented in 179 (77%) patients. The majority, 135 (58%) patients, had intermediate risk (35 [15%] favourable, 34 [15%] poor, 28 [12%] missing information), and 199 (86%) had undergone prior nephrectomy. ccRCC was diagnosed in 188 (81%) patients whereas ncc-histologies were documented in 38 (16%) patients. The latter included papillary type 1 or 2, chromophobe, and others (see Table 1). A sarcomatoid fraction was noted in 10 (4.4%) patients. However, information was unknown/missing for 109 (47%) patients. The most common sites for metastases were the lungs (72%), bones (34%), liver (27%), and adrenal glands (18%). Of the patients, 11 (4.7%) had brain metastases.

### 3.2. Nivolumab Treatment Characteristics and Prior Therapies

Treatment characteristics of nivolumab and prior therapies are shown in Table 2. In 132 (57%) patients, more than 12 months elapsed between primary diagnosis and the start of the first systemic treatment. Median time from initial diagnosis of RCC to the date of the first nivolumab infusion was 32.0 months (range 1.6–363). Median time from last progression to the start of nivolumab therapy was 0.9 months (range 0–65).

Of the patients, 179 (77%) received nivolumab as second-line, 34 (15%) as third-line and 19 (8.2%) as later-line therapy. In most patients, first-line therapy had been pazopanib (*N* = 98; 42%) or sunitinib (*N* = 96; 41%). The most-frequent second-line therapies had been pazopanib, axitinib (*N* = 16; 30% of 53 patients receiving nivolumab as ≥third-line) and everolimus (*N* = 12/53; 23%).

### 3.3. Real-World Effectiveness

**Figure 1:**
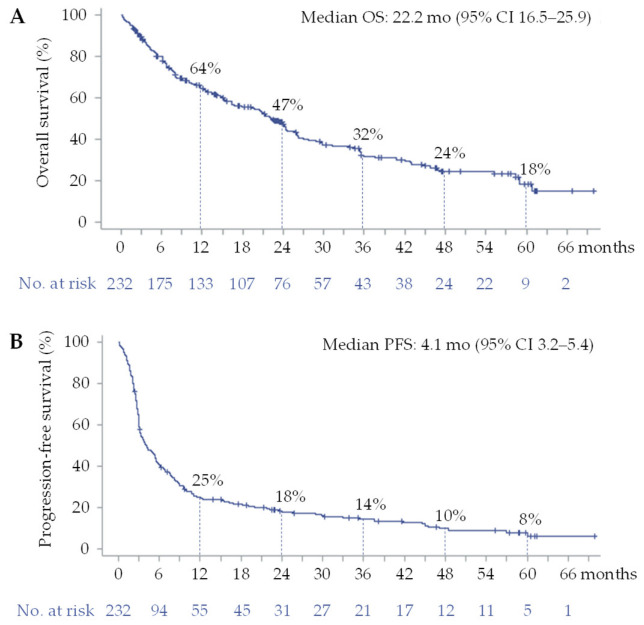
(**A**) Overall survival (OS) and (**B**) progression-free survival (PFS) in the overall population. CI, confidence interval; mo, months; No, number.

Median OS in the overall population was 22.2 months (95% CI 16.5–25.9) with 2-, 3-, and 4-year OS rates of 47%, 32%, and 24%, respectively. Median PFS was 4.1 months (95% CI 3.2–5.4) (Figure 1).

The ORR was 21% (*N* = 49) in the overall population; five patients (2.2%) had complete responses. A further 62 (27%) patients had stable disease, resulting in a DCR of 48% (Table 3). ORR was assessed using imaging criteria in 81 patients and clinical criteria in 164 patients; for a subset of patients, both assessments were available. In NORA, the estimated median treatment duration was 7.4 months (95% CI 6.0–8.8) with an estimated median DOR of 27.9 months (95% CI 15.9-NE). Of the patients, 215 (93%) had discontinued nivolumab treatment by this time, while 17 (7.3%) patients were still receiving nivolumab.

**Figure 2:**
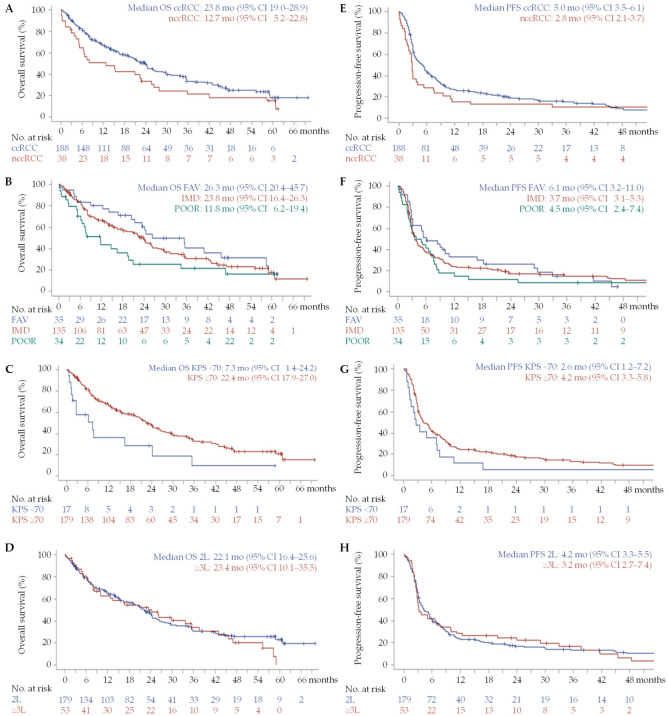
Overall survival (OS) (**A**) in tumour histology subgroups, (**B**) in risk subgroups according to the International Metastatic RCC Database Consortium (IMDC), (**C**) depending on the Karnofsky Performance Score (KPS) and (**D**) per therapy line (second-line [2L] vs. at least third-line [≥3L]). Progression-free survival (PFS) (**E**) in tumour histology subgroups, (**F**) in risk subgroups according to the IMDC, (**G**) depending on the KPS and (**H**) per therapy line. cc, clear cell; CI, confidence interval; FAV, favourable risk; IMD intermediate risk; mo, months; ncc, non-clear cell; No, number; POOR, poor risk; RCC, renal cell carcinoma.

Subgroup analyses revealed lower median OS in patients with nccRCC as compared to those with ccRCC (median OS 12.7 months [95% CI 5.2–22.8] vs. 23.8 months [95% CI 19.0–28.9]; Figure 2A). The ORR was 16% vs. 23% (Table 3). Median OS decreased with increasing IMDC risk, from 26.3 months (95% CI 20.4–45.7) for favourable risk to 23.8 months (95% CI 16.4–26.3) for intermediate risk and to 11.8 months (95% CI 6.2–19.4) for poor-risk patients (Figure 2B). Similarly, median OS was longer in patients with a KPS ≥ 70 than in those with a KPS < 70 (22.4 months [95% CI 17.9–27.0] vs. 7.3 months [95% CI 1.4–24.2]; Figure 2C). No difference in OS was observed between patients receiving nivolumab as second-line therapy and those treated in the third- or later line (22.1 months [95% CI 16.4–25.6] vs. 23.4 months [95% CI 10.1–35.5], Figure 2D). Likewise, OS was not associated with age (<65 and ≥65 years: median OS 22.1 months [95% CI 14.1–35.5] vs. 22.2 months [95% CI 15.5–25.9]; <75 vs. ≥75 years: median OS 24.3 months [95% CI 19.0–27.8] vs. 20.5 months [95% CI 9.1–24.4]).

PFS subgroup analyses yielded similar findings (Figure 2E–H). ORR was lower in patients receiving later lines of therapy (24% for second-line vs. 11% for third- or later lines) and in those with a KPS < 70 (12% vs. 18% for KPS ≥ 70). In contrast, no clear association between IMDC risk category and ORR was observed (23% in favourable risk, vs. 16% in intermediate risk 16%, and 24% in poor risk patients).

### 3.4. Safety Outcomes

A total of 319 trAEs of any grade were reported by 108 (47%) of all patients (Table 4). The most frequent trAE was diarrhea (9.5%), followed by fatigue (6.9%), pruritus (6.5%), and malignant neoplasm progression (6.0%). The most frequent grade 3–4 trAEs were malignant neoplasm progression (3.0%), diarrhea (1.7%), general physical health deterioration (1.7%) and pneumonitis (1.3%). One grade 5 trAE occurred (autoimmune hepatitis). Of the 319 trAE, 41 (13%) led to treatment interruption, and 37 (12%) led to permanent discontinuation.

### 3.5. Quality of Life

**Figure 3:**
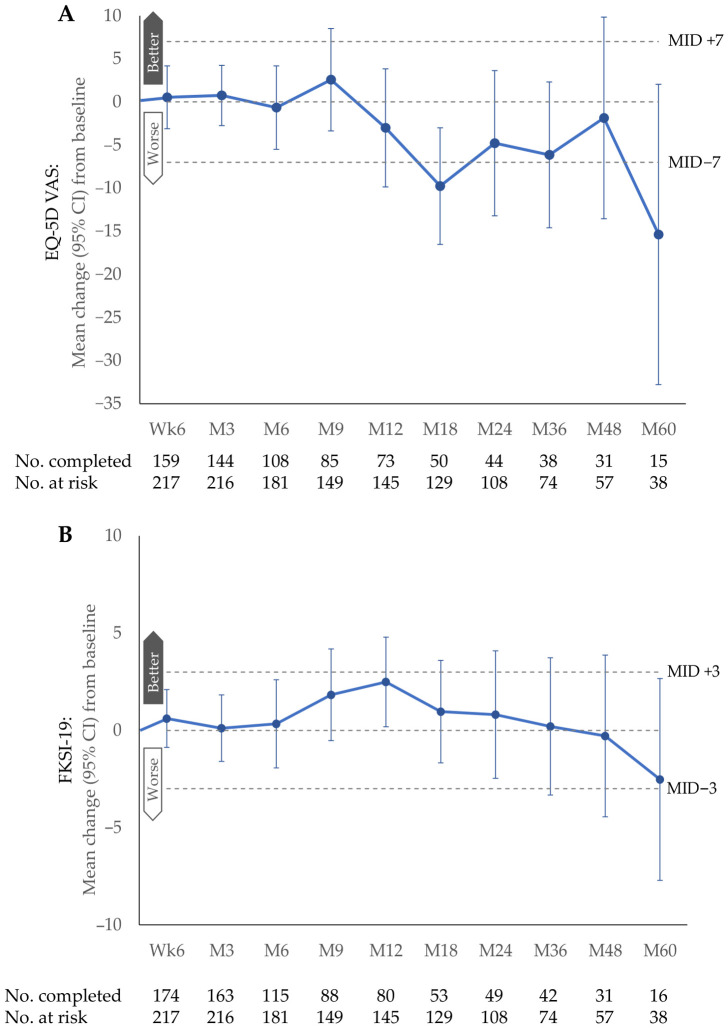
Quality of life. Mean change in score from baseline for (**A**) the European Quality of Life 5 Dimensions (EQ-5D) Visual Analogue Scale (VAS) and (**B**) the Functional Assessment of Cancer Therapy-Kidney Symptom Index-19 (FKSI-19) questionnaires. CI, confidence interval; M, month; MID, minimal important difference; No, number; Wk, week.

Of the 232 patients, 204 (88%) EQ-5D visual analogue scale (VAS) and 216 (93%) FKSI-19 questionnaires were evaluable at baseline. The proportions of completed questionnaires among patients at risk were 73% and 80% for EQ-5D VAS and FKSI-19 at six weeks, respectively. Thereafter, these proportions of completed questionnaires per patients at risk ranged between 67% and 39% for EQ-5D VAS and between 75% and 41% for FKSI-19. PROs are reported and displayed until up to 60 months of follow-up (Figure 3), although the 60-month time point was not interpreted due to the small numbers of patients at risk and responding to questionnaires. The EQ-5D VAS showed no change in quality of life (QoL) during the first year of nivolumab treatment, with an increasing (negative) change from the baseline thereafter. However, the mean change from baseline was below the minimal important difference (MID) only once, at 18 months. The RCC-specific PROs reported with FKSI-19 remained stable during the reporting period. At 9 and 12 months, the (positive) mean change from baseline was most pronounced. However, it never reached the two MIDs during the reporting period.

### 3.6. Subsequent Therapy

Of the 232 patients, 99 (43%) received a subsequent systemic anti-tumour therapy (Table 5). This was mainly cabozantinib (*N* = 61, 26%), everolimus (*N* = 29, 13%), and lenvatinib (*N* = 25, 11%). Of the responders, 36 (73% of 49 responders, 16% of all patients) received subsequent therapy. Data on progression-free survival during this subsequent therapy were not collected. For those patients, the median treatment-free interval, i.e., the time from the last study drug dose to subsequent systemic therapy, was 1.21 weeks (interquartile range 0.21–6.71). Seventeen responders (35% of 49 responders, 7.3% of all patients) remained on therapy at the time of the database lock.

## 4. Discussion

The final analysis of the nivolumab monotherapy cohort of the NORA study demonstrated that nivolumab monotherapy is both effective and generally well tolerated in immune-checkpoint naïve patients with aRCC who have previously undergone treatment, within a real-world clinical setting. The clearly longer median follow-up as compared to our first interim analysis of this cohort (37 months in [9] vs. 76 months [with a minimum follow-up of 62 months] here) is very similar to the one of the latest available data from the pivotal trial CheckMate-025 [6], being 72 months (with a minimum follow-up of 64 months). We therefore assume that the data are as comparable as possible.

Notable differences were observed between the NORA cohort and the nivolumab-treated population in the CheckMate-025 trial. In NORA, the median patient age was higher by nine years (71 vs. 62 years), with 38% of patients aged ≥75 years, compared to only 8% in CheckMate-025. Additionally, 7.3% of patients in NORA had a KPS < 70, a subgroup that was excluded from the randomized CheckMate-025 trial. Nonetheless, two patients (0.5%) in CheckMate-025 were reported with a KPS < 70. Further differences included the presence of ncc-histologies in 16% of NORA patients, whereas only cc-histologies were enrolled in CheckMate-025. In NORA, 4.7% of patients had brain metastases—an exclusion criterion in CheckMate-025—and 8.2% received nivolumab as fourth-line or later therapy, which was also not permitted in the CheckMate-025 trial. Conversely, a smaller proportion of patients in NORA was classified as poor-risk according to IMDC criteria (15% vs. 23% in CheckMate-025), although IMDC risk status was unknown for 12% of the NORA population [5].

Despite a less favourable baseline disease profile in the NORA population (especially ncc-histologies being included, higher IMDC risk, and lower KPS), oncological outcomes were largely comparable between NORA and Checkmate-025. The median PFS in NORA was nearly identical to that reported in Checkmate-025 (4.1 months vs. 4.2 months); the PFS rates after 3 and 4 years were slightly higher in NORA (14% vs. 9% at 3 years; 10% vs. 6% at 4 years). The 5-year PFS rates (8% vs. 5%) were not further interpreted due to the limited number of patients at risk in NORA at this time point (*N* = 9). In contrast, the median OS was somewhat shorter in NORA compared to CheckMate-025 (22.2 vs. 25.8 months), as were the 3- and 4-year OS rates (32% vs. 39% at 3 years; 24% vs. 30% at 4 years). The ORR was similar between the two studies (21% vs. 23%), while the DCR was notably lower in NORA (48% vs. 57%). This difference cannot be attributed to a higher rate of primary progression in the NORA cohort (32% vs. 35%); however, information on best overall response was unavailable for 20% of patients in NORA [6].

Interestingly, despite comparable follow-up times, the NORA study reported a markedly longer DOR compared with CheckMate-025 (27.9 vs. 18.2 months). This finding coincided with a higher proportion of patients with ongoing responses at the data cut-off in NORA (35% vs. 28%). A key methodological difference between the real-world NORA study and the RCT CheckMate-025 lies in the assessment of progression. In CheckMate-025, progression was systematically evaluated in all patients using imaging-based RECIST criteria, whereas in NORA, progression was partly determined by clinical assessment and was therefore less standardized. This heterogeneity in evaluation practices may have contributed to the apparent prolongation of DoR estimates and limits their direct comparability with results from the controlled CheckMate-025 trial. However, there was no evidence suggesting a disadvantage for patients in NORA arising from this real-world practice.

Consistent with the primary analysis of NORA (median follow-up 37 months) [9], ncc-histologies, higher IMDC risk, and a KPS < 70 were associated with shorter median PFS and OS estimates. Compared with ccRCC, in patients with nccRCC, median OS and PFS were approximately halved, and ORR as well as DCR were also numerically lower. Interpretation of these findings is limited by the small number of patients with nccRCC and the biological heterogeneity of this group. Nevertheless, the observed inferior oncological outcomes are consistent with previous reports demonstrating a poorer prognosis for ncc- compared with cc-histologies [21,22,23]. IMDC risk—of which low KPS is one of the evaluation criteria—is an accepted prognostic factor in aRCC. As anticipated, favourable IMDC risk was associated with the highest survival probabilities, in line with the findings from CheckMate-025 [20].

Consistent with the primary analysis, prolonged follow-up showed substantially lower trAE rates in NORA vs. CheckMate-025 (all grades: 47% vs. 81%; grade 3–4: 16% vs. 21%) [6]. In CheckMate-025, the most common grade 3–4 trAEs were fatigue (2.7% vs. 0.4% in NORA), anemia (2.0% vs. 0.9%), and increased alanine and aspartate aminotransferases (1.7% vs. 0% each). In NORA, malignant neoplasm progression (3.0% vs. not reported), diarrhea (1.7% vs. 1%), general physical health deterioration (1.7% vs. not reported), and pneumonitis (1.3% vs. 2%) were the most frequent grade 3–4 trAEs [6]. The lower trAE reporting in NORA likely reflects differences in reporting practices, as the real-world non-interventional setting lacks the stringent, systematic assessments of RCTs like CheckMate-025. Moreover, events such as malignant neoplasm progression and general physical health deterioration are usually not classified as trAEs in RCTs, as they are rarely attributed to study medication, especially in oncology trials. Importantly, no new safety signals were identified, reinforcing the established tolerability of nivolumab in a broader real-world population.

QoL assessed using the EQ-5D VAS remained stable during the first year after treatment initiation, with no meaningful change reported. A gradual decline in QoL was observed up to four years post-treatment, with a clinically meaningful deterioration at 18 months. However, subsequent stabilization questions the relevance of this timepoint. At five years, further decline was noted, but limited data (*N* = 15) reduce interpretability. Kidney cancer-related QoL measured by FKSI-19 showed no clinically meaningful deterioration up to five years. These findings are consistent with CheckMate-025 results, which reported no deterioration—and a trend toward improvement—in QoL over the reported 2.5-year period using the Functional Assessment of Cancer Therapy Kidney Symptom Index–Disease-Related Symptoms (FKSI-DRS [24]) questionnaire [6]. The questionnaires differed between the two studies, with the FKSI-19 including all items of the FKSI-DRS as well as additional questions. This difference should be taken into account when making comparisons.

In the NORA study, a significantly lower proportion of patients received subsequent systemic anti-tumour therapy compared to CheckMate-025 (43% vs. 67%). Among responders who discontinued treatment and subsequently received further therapy, the median interval from the last dose of the study drug to initiation of subsequent systemic therapy was 7.9 weeks in CheckMate-025 and 1.2 weeks in NORA. The shorter interval to subsequent therapy observed in NORA versus CheckMate-025 might reflect more flexible, individualized treatment decisions in the real-world setting in Germany.

In CheckMate-025, the most common subsequent systemic therapies in the nivolumab arm were everolimus (35%), axitinib (33%), cabozantinib (14%), and pazopanib (12%). In NORA, cabozantinib (26%), everolimus (13%), and lenvatinib (11%) were the most frequently used [6]. Differences in subsequent therapies between CheckMate-025 (recruitment 2012–2014) and NORA (2016–2018) reflect changes in approval status and treatment availability. During CheckMate-025, everolimus and axitinib were standard second-line options, while cabozantinib and lenvatinib were less established. By the time of NORA, cabozantinib had become widely approved and more commonly used, explaining its higher usage, whereas everolimus and lenvatinib were less frequent. These findings highlight the influence of evolving therapeutic landscapes on real-world treatment patterns.

It should be noted that additional real-world studies have investigated the effectiveness of nivolumab in second- or later line aRCC, including the French WITNESS study [25] and a study conducted by the Italian Nivolumab Renal Cell Cancer Early Access Programme (IEAP) group [26,27]. However, both studies reported a median follow-up of approximately one year and were therefore not included in the present discussion. For WITNESS, only interim results are available so far. The final data with longer follow-up have to be awaited.

Today, ICI-based combinations represent the standard first-line treatment for aRCC. However, TKI monotherapy is still an appropriate option for IMDC low-risk patients and recommended by current guidelines for this group [28,29,30]. Consequently, nivolumab monotherapy remains a strongly recommended second-line treatment following TKI monotherapy.

The NORA study has several limitations, primarily related to data collection. The absence of centralized pathology review and standardized follow-up, e.g., with regard to frequency and extent of assessments including imaging, represent significant constraints. Despite these limitations, the findings indicate that nivolumab demonstrates a consistent treatment effect alongside a favourable tolerability profile.

## 5. Conclusions

The final analysis of the NORA study confirms that nivolumab monotherapy is effective, safe, and generally well tolerated in a real-world population of previously treated patients with aRCC. These findings were observed despite the inclusion of patients with ncc-histologies, poorer performance status, older age, and a broader range of prior treatment lines compared to the pivotal CheckMate-025 trial, with, however, no apparent impact of the latter two factors on oncological outcomes. Despite limitations inherent to real-world data, these results support the robust and durable benefit of nivolumab in a broad-spectrum, routine clinical setting. Its outcomes align with those of CheckMate-025 and expand evidence to patient subgroups excluded from that study.

## Figures and Tables

**Table 1:** Baseline characteristics for patients and disease for patients treated with nivolumab after at least one prior systemic therapy (NORA) and patients from CheckMate 025 [5,6,20].

Parameter	NORA (*N* = 232)	CheckMate 025 (*N* = 410)
Median age, years (range)	71 (44–86)	62 (23–88)
Age category, *N* (%)		
<65 years	73 (31)	257 (63)
≥65, <75 years	70 (30)	119 (29)
≥75 years	89 (38)	34 (8.3)
Sex, *N* (%)		
Male	166 (72)	315 (77)
Female	66 (28)	95 (23)
Karnofsky Performance Score, *N* (%)		
<70	17 (7.3)	2 (0.5)
70	18 (7.8)	22 (5.4)
80	63 (27)	110 (27)
90	53 (23)	150 (37)
100	45 (19)	126 (31)
Missing	36 (16)	-
IMDC risk, *N* (%)		
Favourable	35 (15)	55 (13)
Intermediate	135 (58)	242 (59)
Poor	34 (15)	96 (23)
Missing	28 (12)	-
Histology Subtype at initial diagnosis, *N* (%)		
Clear cell	188 (81)	410 (100)
Non-clear cell	38 (16)	-
Papillary type 1	11 (4.7)	-
Papillary type 2	13 (5.6)	-
Chromophobe	4 (1.7)	-
Other	10 (4.3)	-
Missing	6 (2.6)	-
Location of metastases, *N* (%) ^1^		
Lung	166 (72)	278 (68)
Bone	73 (34)	76 (19)
Liver	63 (27)	100 (24)
Adrenal glands	42 (18)	n.a.
Lung and/or malignant pleural effusion	18 (7.8)	n.a.
Brain	11 (4.7)	- ^2^
Peritoneal and/or malignant ascites	10 (4.3)	n.a.
No metastases	2 (0.9)	n.a.
Other	100 (43)	n.a.
Sarcomatoid fraction, *N* (%)		
Yes	10 (4.3)	n.a.
No	113 (49)	n.a.
Unknown	107 (46)	n.a.
Missing	2 (0.9)	n.a.
Nephrectomy, *N* (%)		
Yes	199 (86)	364 (89)
No	29 (12.5)	46 (11)
Missing	4 (1.7)	-

Percentages may not add up to 100% due to rounding imprecision. ^1^ Multiple locations per patient possible. ^2^ Patients with metastases to the central nervous system were excluded from CheckMate-025. MSKCC: Memorial Sloan Kettering Cancer Center; IMDC: International Metastatic RCC Database Consortium; n.a.: not available.

**Table 2:** Prior systemic tumour therapy and nivolumab treatment characteristics.

Parameter	NORA (*N* = 232)
Median disease duration at baseline, months (range)	32.0 (1.6–363)
Study treatment line for nivolumab, *N* (%)	
Second-line	179 (77)
Third-line	34 (15)
Fourth- or later line	19 (8.2)
Prior first-line, *N* (%) ^1^	
Pazopanib	98 (42)
Sunitinib	96 (41)
Everolimus, temsirolimus	14 (6.0)
Other TKI monotherapy	11 (4.7)
Other	16 (6.9)
Prior second-line, *N* (%) ^1,2^	
Pazopanib	16 (30)
Axitinib	16 (30)
Everolimus	12 (23)
Sunitinib	5 (9.4)
Other TKI monotherapy	7 (13)
Other	3 (5.7)
Prior third-line, *N* (%) ^1,3^	
Everolimus	5 (26)
Axitinib	3 (16)
Sunitinib	4 (21)
Other TKI monotherapy	6 (32)

^1^ Multiple records per patient possible. ^2^ Percentages based on 53 patients classified as third- or later line. ^3^ Percentages based on 19 patients classified as fourth- or later line. TKI, tyrosine kinase inhibitor.

**Table 3:** Antitumor activity: Best overall response to nivolumab monotherapy after at least one prior treatment line, based on clinical or RECIST assessment (response assessed at site level according to clinical practice). Reported for NORA intention-to-treat (ITT) population, clear-cell-(cc-) and non-clear-cell-(ncc-) histology of renal cell carcinoma (RCC), and Checkmate-025 ITT [6].

Parameter	ITT (*N* = 232)	ccRCC (*N* = 188)	nccRCC (*N* = 38)	Checkmate-025 (*N* = 410)
Objective response rate, *N* (%)	49 (21)	43 (23)	6 (16)	94 (23)
Disease control rate, *N* (%)	111 (48)	92 (49)	15 (39)	234 (57)
Best overall response, *N* (%)				
Complete response	5 (2.2)	4 (2.1)	1 (2.6)	4 (1)
Partial response	44 (19)	39 (21)	5 (13)	90 (22)
Stable disease	62 (27)	49 (26)	9 (24)	140 (34)
Progressive disease	74 (32)	59 (31)	14 (37)	142 (35)
Missing	47 (20)	37 (20)	9 (24)	34 (8)

**Table 4:** Treatment-related adverse events (trAEs) in patients treated with nivolumab monotherapy for advanced renal cell carcinoma after at least one prior treatment line. All grades (if incidence ≥ 5%) and grade 3–4 (all events). One treatment-related death occurred (autoimmune hepatitis). *N* = 232. Patients could experience several trAEs. Data are reported as preferred terms according to Common Terminology Criteria for Adverse Events (CTCAE) Version 4.0.

Treatment-Related Adverse Events (trAEs), *N* (%)	All Grades	Grade 3–4
All	108 (46.6)	36 (15.5)
Diarrhea	22 (9.5)	4 (1.7)
Fatigue	16 (6.9)	1 (0.4)
Pruritus	15 (6.5)	0
Malignant neoplasm progression	14 (6.0)	7 (3.0)
Pneumonitis	6 (2.6)	3 (1.3)
Dyspnoea	6 (2.6)	1 (0.4)
General physical health deterioration	4 (1.7)	4 (1.7)
Anemia	4 (1.7)	2 (0.9)
Psoriasis	4 (1.7)	1 (0.4)
Colitis	3 (1.3)	2 (0.9)
Acute kidney injury	3 (1.3)	2 (0.9)
Edema peripheral	3 (1.3)	1 (0.4)
Liver disorder	2 (0.9)	1 (0.4)
Pneumonia	2 (0.9)	1 (0.4)
Gamma-glutamyltransferase increased	2 (0.9)	1 (0.4)
Arrhythmia	1 (0.4)	1 (0.4)
Autoimmune hepatitis	1 (0.4)	1 (0.4)
Hypersensitivity	1 (0.4)	1 (0.4)
Clostridium difficile colitis	1 (0.4)	1 (0.4)
Gastroenteritis	1 (0.4)	1 (0.4)
Blood lactate dehydrogenase increased	1 (0.4)	1 (0.4)
Blood potassium increased	1 (0.4)	1 (0.4)
C-reactive protein increased	1 (0.4)	1 (0.4)
Bone pain	1 (0.4)	1 (0.4)
Disturbance in attention	1 (0.4)	1 (0.4)
Chronic kidney disease	1 (0.4)	1 (0.4)
Pleural effusion	1 (0.4)	1 (0.4)
Lichenoid keratosis	1 (0.4)	1 (0.4)
Hypertension	1 (0.4)	1 (0.4)

**Table 5:** Subsequent anti-tumour therapy.

Parameter	NORA (*N* = 232)
Any subsequent treatment, *N* (%) ^1^	99 (43)
Cabozantinib	61 (26)
Everolimus	29 (13)
Lenvatinib	25 (11)
Axitinib	15 (6)
Pazopanib	9 (4)
Sunitinib	8 (3)
Nivolumab	6 (3)
Tivozanib	5 (2)
Other	7 (3)

^1^ Multiple records per patient possible.

## Data Availability

Deidentified trial data are accessible per request addressed to the corresponding author (marc-oliver.grimm@med.uni-jena.de) or the sponsor. The sponsor will thoroughly consider providing qualified scientific researchers access to deidentified data and clinical study reports from NORA for the purpose of conducting legitimate scientific research. The sponsor is obligated to protect the rights and privacy of trial participants and, as such, has a procedure in place for evaluating and fulfilling requests for sharing clinical trial data with qualified external scientific researchers. In line with data privacy legislation, submitters of approved requests must enter into a standard data-sharing agreement with the sponsor before data access is granted. There are circumstances that might prevent the sponsor from sharing requested data, including country or region-specific regulations. If the request is declined, it will be communicated to the investigator.

## Abbreviations

The following abbreviations are used in this manuscript:

AE	Adverse events
aRCC	Advanced renal cell carcinoma
BOR	Best overall response
ccRCC	Clear cell renal cell carcinoma
CI	Confidence interval
DCR	Disease control rate
DOR	Duration of response
EMA	European Medicines Agency
EQ-5D VAS	European Quality of Life 5 Dimensions questionnaire—Visual analogue scale
EQ-5D-3L	European Quality of Life 5 Dimensions 3 Level Version
FKSI-19	Functional Assessment of Cancer Therapy—Kidney Symptom Index-19
FKSI-DRS	Functional Assessment of Cancer Therapy Kidney Symptom Index–Disease-Related Symptoms
HR	Hazard ratio
IEAP	Italian Nivolumab Renal Cell Cancer Early Access Programme
IMDC	International Metastatic Renal Cell Carcinoma Database Consortium
ITT	Intention-to-treat
KPS	Karnofsky performance score
MID	Minimal important difference
MSKCC	Memorial Sloan Kettering Cancer Center
n.a.	Not available
nccRCC	Non-clear cell renal cell carcinoma
NCI CTCAE	National Cancer Institute Common Terminology Criteria for Adverse Event
NE	Not Estimable
NIS	Non-interventional study
NORA	NivOlumab in Renal cell cArcinoma
ORR	Objective response rate
OS	Overall survival
PD-1	Programmed cell death protein 1
PD-L1	Programmed death-ligand 1
PFS	Progression-free survival
PRO	Patient-reported outcomes
QoL	Quality of life
RCT	Randomized clinical trial
RECIST	Response Evaluation Criteria in Solid Tumors
TKI	Tyrosine kinase inhibitor
trAE	Treatment-related adverse events
